# A fatal case of metastatic gastric adenocarcinoma mimicking cirrhosis

**DOI:** 10.4322/acr.2021.325

**Published:** 2021-09-03

**Authors:** Janet Marie Basinger, Jonathan Tucci, Meghan Elizabeth Kapp

**Affiliations:** 1 Vanderbilt University Medical Center, Department of Pathology, Microbiology and Immunology, Nashville, TN, USA

**Keywords:** Pseudocirrhosis, Adenocarcinoma, Gastric

Pseudocirrhosis is a rare entity that closely mimics cirrhosis clinically and radiographically but lacks the histopathologic features of cirrhosis.^1^,^2^ It is most commonly described in metastatic breast carcinoma and associated chemotherapeutic regimens; however, it has been reported with metastatic carcinoma of other origins, including metastatic esophageal squamous cell carcinoma and gastric adenocarcinoma following chemotherapy.^3^,^4^ It is associated with significant morbidity and mortality, even after initiation of appropriate treatment.^1^ The macroscopic findings differ from classic macronodular cirrhosis, as is demonstrated by lesional, desmoplastic nodules rather than abnormal liver parenchyma with surrounding bridging fibrosis forming nodules. The microscopic findings vary, but generally present as one of two variations. The first is a pattern of nodular regenerative hyperplasia with capsular retraction and is associated with chemotherapy regimens or possibly chemotherapeutic response to metastatic tumor.^2^ The second histopathologic pattern is infiltration by metastatic carcinoma with a desmoplastic response to the tumor cells, as seen in this case.

**Figure 1:**
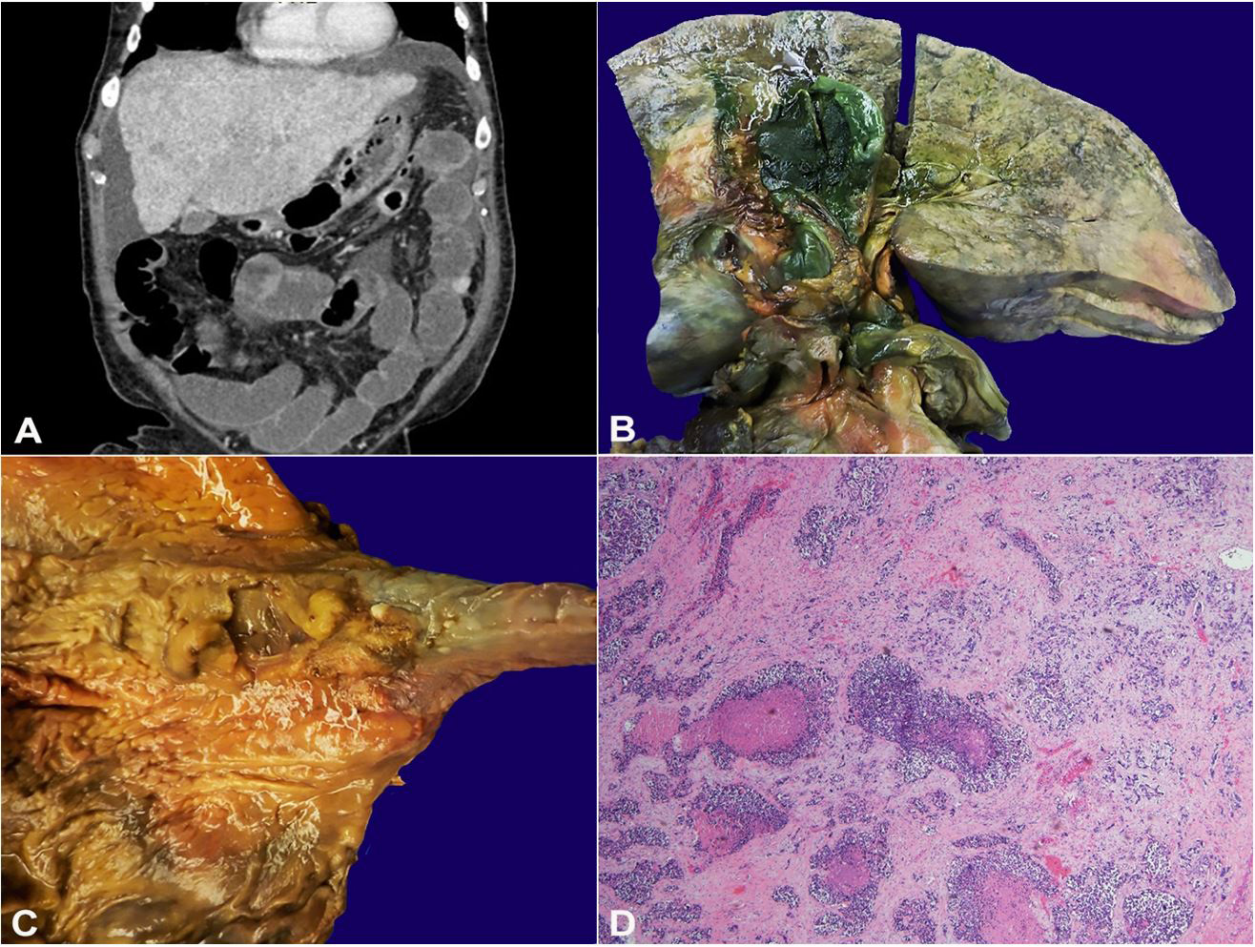
**A** – Non-contrast enhanced CT of the abdomen and pelvis demonstrates cirrhotic morphology of the liver with hypoenhancing lesions and large volume ascites; **B** – Posterior aspect of the liver, cut surface, showing replacement of the normal liver parenchyma by tan-white, ill-defined, fibrotic nodules, and gallbladder with a slightly thickened wall; **C** – Esophagus and stomach with indurated plaque-like mass centered below the gastroesophageal junction; **D** – Photomicrograph of the liver demonstrating replacement of normal parenchyma by metastatic adenocarcinoma with necrosis and desmoplasia without evidence of background fibrosis or hepatitis (H&E, 400x).

The pictures belong to a 71-year-old man who presented with decompensated cirrhosis of unknown etiology. Cirrhosis and sequelae of portal hypertension were originally diagnosed by computed tomography (Figure 1A). His clinical course was complicated by hepatic encephalopathy and large volume ascites with concern for spontaneous bacterial peritonitis, and he passed away shortly after transition to palliative care.

Consent for a full autopsy was obtained and the findings are shared with IRB approval (VUMC IRB 211285). Postmortem examination revealed an enlarged liver with capsular nodularity and marked replacement of hepatic parenchyma by dense fibrotic nodules on cut surface (Figure 1B). Additionally, there was a 2.5 cm indurated, plaque-like mass centered below the gastroesophageal (GE) junction (Figure 1C). Microscopic sections of the GE junction mass showed poorly differentiated adenocarcinoma with neuroendocrine and signet-ring features. Histologic examination of the liver showed marked replacement of the liver parenchyma by metastatic adenocarcinoma without evidence of background fibrosis or hepatitis (Figure 1D). Furthermore, metastatic adenocarcinoma was present in the lungs, thoracic and abdominal lymph nodes, and gallbladder wall. Thus, the cause of death was liver failure due to metastatic gastric adenocarcinoma.
